# Editorial: Bioactive natural products for health: isolation, structural elucidation, biological evaluation, structure-activity relationship, and mechanism

**DOI:** 10.3389/fchem.2024.1428905

**Published:** 2024-05-22

**Authors:** Qinge Ma, Hui Xiao, Huilian Huang, Chunsu Yuan, Rongrui Wei

**Affiliations:** ^1^ Key Laboratory of Modern Preparation of Traditional Chinese Medicine of Ministry of Education, Research Center of Natural Resources of Chinese Medicinal Materials and Ethnic Medicine, Jiangxi University of Traditional Chinese Medicine, Nanchang, China; ^2^ Department of Anesthesia and Critical Care, Tang Center for Herbal Medicine Research, Committee On Clinical Pharmacology and Pharmacogenomics, Pritzker School of Medicine, The University of Chicago, Chicago, IL, United States

**Keywords:** natural products, bioactivity, phytochemistry, pharmacology, mechanism

Natural products in medicinal plants are an important source of new drug discovery because they contain thousands of active ingredients with novel structures and unique diversities. Although a large number of natural active products are isolated from medicinal plants, the pharmacodynamic material bases of some new natural products are not clear, and the structure-activity relationship and mechanism of action are not clarified (Mathias, 2017; Bhawna and Ashwani, 2021). Above factors have seriously hindered the discovery of new drugs from medicinal plants. Consequently, the discovery of new active natural products from medicinal plants is of great practical significance for the development of new drugs. We are deeply privileged to compile this research topic of Frontiers in Chemistry dedicated to “Bioactive Natural Products for Health: Isolation, Structural Elucidation, Biological Evaluation, Structure-Activity Relationship, and Mechanism”.

The study by Pires et al. discussed about the critical evaluation of clinical trials of phytates. Phytates are a type of organophosphorus compound produced in terrestrial ecosystems by plants. This updated review aimed to summarize the current data on the results of clinical trials of phytates on human health, highlighting both beneficial and undesirable effects. Study results showed that phytate could have beneficial health effects such as anti-oxidant, anti-cancer potential and reduction of pathological calcifications in blood vessels and organs; but also, negative effects by reducing the absorption of minerals important for maintaining the homeostasis of the human body. Phytates might a potential natural source for health benefits according to these recent results derived from recent clinical studies.

Jia et al. investigated the synthesis and hypoglycemic activity of quinoxaline derivatives. In this study, a new series of quinoxalinone derivatives (5a-5p, 6a-6n) was designed and its hypoglycemic activity was evaluated. The results showed that compounds 5i and 6b exhibited stronger hypoglycemic effects than the lead compounds and were comparable to the positive control Pioglitazone. 5i and 6b might exert hypoglycemic effects by alleviating cellular OS and modulating the interactions among GLUT4, SGLT2, and GLUT1 proteins. The alleviating cellular OS of compound 6b was better than that of 5i, and 6b was found to bind better than 5i for most of the screening targets. In summary, compound 6b was a potential lead compound with hypoglycaemic activity.

Chaudhary et al. discussed about oxidative stress, free radicals and anti-oxidants: potential crosstalk in the pathophysiology of human diseases. To counteract the condition of oxidative stress, synthetic anti-oxidants must be provided from external sources to supplement the antioxidant defense mechanism internally. Because of their therapeutic potential and natural origin, medicinal plants have been reported as the main source of natural anti-oxidants phytocompounds. Some non-enzymatic phytocompounds such as flavonoids, polyphenols, and glutathione, along with some vitamins have been reported to possess strong anti-oxidant activities *in vivo* and *in vitro* studies. Thus, the review described the overview of oxidative stress-directed cellular damage and the unction of dietary anti-oxidants in the management of different diseases. The therapeutic limitations in correlating the anti-oxidant activity of foods to human health were also discussed.

The study by Li et al. demonstrated rutaecarpine was a natural pentacyclic indolopyridoquinazolinone alkaloid first isolated from one of the most famous traditional Chinese herbs. Accumulating pharmacological studies showed that rutaecarpine possessed a wide range of pharmacological effects through different mechanisms. In this regard, the modification of rutaecarpine aimed at seeking its derivatives with better physicochemical properties and more potency had been extensively studied. These derivatives exhibited diverse pharmacological activities, including anti-inflammatory, anti-atherogenic, anti-Alzheimer’s disease, anti-tumor, and anti-fungal activities via a variety of mechanisms, such as inhibiting COX-2, AChE, PDE4B, PDE5, or Topos. From this perspective, this paper provided a comprehensive description of rutaecarpine derivatives by focusing on their diverse biological activities. This review aimed to give an insight into the biological activities of rutaecarpine derivatives and encourage further exploration of rutaecarpine.

Amaghnouje et al. aimed to evaluate the bioavailability of a polyphenolic extract obtained from *Origanum majoran*a L. leaves, as well as its anti-epileptic activity and its potential mechanism of action. Both compounds tested showed low bioavailability in unchanged form. However, the tested extract showed an anti-convulsant effect due to the considerably delayed onset of seizures in the pilocarpine model at a dose of 100 mg/kg. The molecular docking proved a high-affinity interaction between the caffeic acid and quercetin with the N-methyl-D-aspartate receptor. Taken together, *O. majoran*a L. polyphenols demonstrated good anti-epileptic activity, probably due to the interaction of quercetin, caffeic acid, or their metabolites with the N-methyl-D-aspartate receptor receptor.

Nawaz et al. studied biochemical, structural characterization and *in vitro* evaluation of anti-oxidant, anti-bacterial, cytotoxic, and anti-diabetic activities of nanosuspensions of *Cinnamomum zeylanicum* bark extract. This study was designed to evaluate the bioactivities, biochemical characterization, and bioavailability of freshly prepared nanosuspensions of *C. zeylanicum*. Structural and biochemical characterization of *C. zeylanicum* and its biological activities, such as anti-oxidants, anti-microbials, anti-glycation, *α*-amylase inhibition, and cytotoxicity was performed using FTIR and HPLC. *C. zeylanicum* extract and nanosuspensions showed TPCs values of 341.88 and 39.51 mg GAE/100 g while showing TFCs as 429.19 and 239.26 mg CE/100g, respectively. DPPH inhibition potential of *C. zeylanicum* extract and nanosuspension was 27.3% and 10.6%, respectively. Biofilm inhibition activity revealed that bark extract and nanosuspension showed excessive growth restraint against *Escherichia coli*, reaching 67.11% and 66.09%, respectively. The *α*-amylase inhibition assay of extract and nanosuspension was 39.3% and 6.3%, while the antiglycation activity of nanosuspension and extract was 42.14% and 53.76%, respectively. Extracts and nanosuspensions showed maximum hemolysis at 54.78% and 19.89%, respectively. Results indicated that nanosuspensions possessed anti-diabetic, anti-microbial, anti-cancer, and anti-oxidant properties.

Wang et al. investigated on-line identification of the chemical constituents of *Polygoni multiflori* Radix by ultra-high-performance liquid chromatography-quadrupole time-of-flight mass spectrometry (UHPLC-Q-ToF MS/MS). Three solvents of different polarities (water, 70% ethanol, and 95% ethanol solution) were used to extract the compounds from PMR. Extracts were analyzed and characterized by UHPLC-Q-ToF MS/MS in the negative-ion mode. 152 compounds were detected and identified: 50 anthraquinones, 33 stilbene derivatives, 21 flavonoids, seven naphthalene compounds, and 41 other compounds. Eight other compounds were reported for the first time in the PMR-related literature, and eight other compounds were potentially new compounds. This study lays a solid foundation for the screening of toxicity and quality-control indicators of PMR.

Yu et al. found that two new pyranone derivatives phomapyrone A and phomapyrone B, one new coumarin 11*S*,13*R*-(+)-phomacumarin A, three known pyranones, together with three known amide alkaloids fuscoatramides A-C, as well as 9*S*, 11*R*-(+)-ascosalitoxin were isolated from the endophytic fungus *Phoma* sp. YN02-P-3, which was isolated from the healthy leaf tissue of a Paulownia tree in Yunnan Province, China. Their structures were elucidated using extensive NMR spectroscopic and HR-ESI-MS data and by comparing the information with literature data. In addition, all compounds were tested for their cytotoxicity activity against human tumor cell lines, and the results showed that three new compounds showed moderate inhibitory activity against the HL-60 cell line with IC_50_ values of 31.02, 34.62, and 27.90 μM, respectively.

Yu et al. made a chemical investigation on the kiwi endophytic fungus Bipolaris sp. Resulted in the isolation of eight new terpenoids and five known analogues, including five novel sativene sesquiterpenoids containing three additional skeletal carbons, while two novel compounds are rare dimers. Among them, three compounds were sesterterpenoids that had been identified from this species for the first time. Two new compounds showed anti-bacterial activities against kiwifruit canker pathogen *Pseudomonas syringae* pv. Actinidiae (Psa) with MIC values of 32 and 64 μg/mL, respectively.
